# Barriers preventing women and girls from accessing the eye care they need

**Published:** 2025-03-07

**Authors:** Preeti Dhingra

**Affiliations:** 1 Head of Sustainability: Mission for Vision, Mumbai, India.

**To improve access, we must first understand the impact of social expectations on women and girls.**

**Figure:**
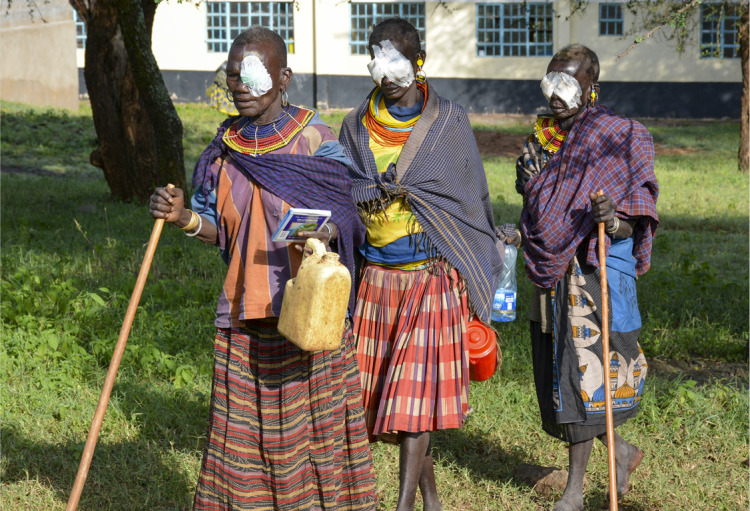
Three older women had to travel for two days, tied together for safety, to receive sight-restoring cataract surgery. KENYA

© MARK MAINA CC BY-NC-SA 4.0

Barriers such as cost, distance, and the acceptability of eye care services prevent both women and men from receiving the eye care they need. However, these barriers are often more pronounced for women due to social or cultural expectations. Women also face additional, internalised barriers.^1,2^

## Social and cultural barriers

- Almost everywhere around the world, women are expected to be the primary caregivers, doing unpaid domestic work and caring for children and elderly or vulnerable family members.
- Women tend to have less control over family finances as their caring or domestic work is unpaid and undervalued, and/or because they cannot undertake paid work outside the home (due to lack of support, or their domestic and caring responsibilities). As a result, women tend to have less decision-making power in the family, and less say in how money is spent.
- Women may be actively discouraged or even forbidden from travelling alone due to social and cultural expectations or safety concerns.
- In some cultures, women may not be allowed to speak to men, including health workers, without a male family member being present.

## Distance and cost

Due to these social and cultural barriers, travelling long distances for eye care, and waiting in line for a long time, can be a major barrier. For example:

- Women may have to wait until a male family member can accompany them, and these men may lose wages if they take time away from work.
- Women either need to find someone to take on their caring responsibilities while they travel, or they need to bring their dependents (e.g., children) with them, increasing travel costs.
- Women's lack of financial independence, plus the attitudes of male heads of household, can either support or discourage women from seeking eye health care.^3^ This is particularly challenging in countries without national health insurance or similar social safety nets.

## Internalised and other barriers

- Social and cultural expectations of women can lead women to prioritise their family's needs over their own need for eye care. As a result, they experience higher rates of unemployment and dependency, perpetuating gender inequity.^4^
- Women with vision problems may feel that they are a burden. Social stigma about blindness or disability can be so deeply ingrained in the subconscious of women who are blind or have vision impairment that shame may prevent them from seeking help.^5,6^
- A decline in vision is often viewed as an inevitable consequence of ageing and women affected by vision loss are less likely to have social support in a family to seek care.^3^
- Women's rates of literacy are often lower than men's (because their education was not prioritised), especially among older people. Consequently, women can be unaware of information regarding vision loss or other eye health conditions and may be less likely to know about the possibility of treatment or where to receive it.

A case study from India^7^ found that women in India required more preparation before accessing medical services due to cultural reasons, including: waiting for permission to travel, waiting for finances to be allocated, having an accompanying person, and preparing for time away from household and child care tasks expected of them.

It is also important to understand barriers from the perspective of women who experience intersectional marginalisation (for instance, those who are also older, have a disability, live in rural areas, are socio-economically disadvantaged, and/or are members of an ethnic minority group).

It is vital that everyone involved in providing eye care understands that social and cultural expectations have an impact on the way women and girls are able to access to eye care. It is our responsibility to challenge our own perceptions and biases to ensure eye health information and services reach women and girls, especially those who experience intersecting forms of discrimination.
